# Evaluation of the Effect of Morphine and Imiquimodon Expression of *TLR2* and *TLR4* from Lesion RNA Extracted from BALB/c Mice Infected with *Leishmania major*

**Published:** 2019

**Authors:** Parisa Ebrahimisadr, Fatemeh Ghaffarifar, John Horton, Abdolhossein Dalimi, Zohreh Sharifi

**Affiliations:** 1. Department of Parasitology and Entomology, Faculty of Medical Sciences, Tarbiat Modares University, Tehran, Iran; 2. Tropical Projects, Hitchin, United Kingdom; 3. Blood Transfusion Research Center, High Institute for Education and Research in Transfusion Medicine, Tehran, Iran

**Keywords:** *Leishmania major*, Lesions, Mice, Morphine, Toll-like receptors

## Abstract

**Background:**

Toll-Like Receptors (TLRs) are the cause of phagocytosis activation and destruction of the infection agents. In addition, new evidences support the idea that TLRs play a vital role in starting the acquired immunity reactions.

**Methods:**

In this study, it has been attempted to infect the BALB/c mice with Leish*m*ania *m*ajor (*L. major*) and treat them using morphine and imiquimod; then the expressions of *TLR2*,4 from treated lesion were studied by using Real-Time PCR method. Treatment with morphine 1 *mg/kg*, imiquimod 5% and nalmefene 1 *mg/kg* began four weeks after the challenge. After treatment period, half of the mice of each group were killed and their lesions were isolated for RNA extraction and making cDNA. For the rest of mice, lesion size was measured weekly.

**Results:**

The results showed increase of expression of *TLR2* gene among all treated groups relative to the control, and the difference was significant (p<0.05). The expression of *TLR4* gene only was reduced in groups under treatment with morphine and morphine plus nalmefene relative to the control group and in the other groups increased. The highest expression of *TLR2* was seen in the group treated by glucantime (p<0.0001).

**Conclusion:**

However, in this study it was found that despite decreasing the size of lesion in all treated groups, expression of *TLR4* in the morphine, nalmefene, morphine plus nalmefene treated groups compared to the control group was decreased. Therefore, morphine may have a different function mechanism in treatment of the Leishmaniasis with the *L. major*.

## Introduction

Leishmaniasis is a complex disease caused by various species of Leishmania, with a variety of clinical features 1. Innate immunity system is capable of detecting the protected microbial structures or microbial metabolism products ([Pathogen Associated Molecular Patterns (PAMPs)], through a set of encoded receptors, called Pattern Reception Recognition (PRR) model 2. PRRs of the innate immunity system, specifically Toll-Like Receptors (TLRs) are responsible for the acute inflammatory responses, through induction of anti-microbial genes and inflammatory cytokines. TLRs play a crucial role in innate and acquired immunity systems as well as increase of phagocytosis and parasite killing process 3. Imiquimod components stimulate the innate immunity systems directly and lead to production of cytokines 4. Morphine function is caused by opioid receptor's intermediates 5. The first is a selective drug for the treatment of Leishmaniasis of antimony compounds. However, these drugs also have side effects such as drug resistance and relapse after treatment 6. Nalmefeneis an antagonist of opioid and acts as an inhibitor for opioid receptors and could increase intracellular pathogenicity 7. There are a few studies on *TLR2* and *TLR4* in animal model and the probable relation with Leishmania infections 8. In this study, it has been attempted to infect the BALB/c mice with Leish*m*ania *m*ajor (*L. major*) and treat them using morphine and imiquimod; then the expression levels of TLRs 2 and *TLR4* were evaluated in treated lesions.

## Materials and Methods

The *L. major* strain (MRHO/IR/75/ER) was stored in Parasitology Department, Faculty of Medical Sciences, Tarbiat Modares University. Promastigotes of the *L. major* were cultured in RPMI 1640 (Gibco, US) media contained FBS 10% (Fetal Bovine Serum, Gibco, US) with 100 *IU*/*ml* penicillin and 100 μg/*m*l streptomycin in 24–26*°C* incubator. Female BALB/c mice (6–8 weeks) were purchased from Pasteur Institute of Iran. The BALB/c mice were kept under standard light, food and water conditions. When the parasite entered the stationary phase, 2×10^6^promastigotes were injected to the tail base of BALB/c mice 9. Treatment was started after inoculation and continued for four weeks. Next, 1 *mg/kg* morphine sulfate was used once a week (Temad Co.). Imiquimod (5% Aldara cream, MEDA Co.) and nalmefene (Selincro Co.) were prepared according to the instruction of the producer. Glucantime (Sanofi-Aventis, France) was applied as the positive control. After ulceration, ten BALB/c mice from each group were treated by various drugs for 4 weeks according to the following classification:

1. Control (Untreated mice);
2. Treated with morphine (1 *mg/kg*, once a week for 4 weeks after the injury**),** subcutaneous injection (M);
3. Treated with nalmefene (1 *mg/kg*, once a week for four weeks, intraperitoneal injection) (N);
4. Treated with glucantime (20 *mg/kg* daily for 28 days, intramuscular injection) (G);
5. Treated with imiquimod (5% imiquimod cream, Aldara, topical treatment) (I);
6. Treated with nalmefene and morphine (N+M);
7. Treated with nalmefene and glucantime (N+G);
8. Treated with imiquimod and morphine (I+M) and
9. Treated with imiquimod and glucantime (I +G).

After treatment period, half of the mice of each group were killed and their lesion was isolated in plates; they were homogenized, and RNA was extracted according to Qiagen Kit protocol (RNeasy Mini Kit, Qiagen Company).

The amount of the extracted RNA was measured using Biophotometer device. Next, 1 *μg* of the RNA was used to make cDNA according to the Qiagen Kit protocol (Quanti Tect Reverse Transcription Kit, Qiagen company). In this study, the QIAGEN Master cycler was used to test the real-time PCR quality. For analysis of expression of *TLR2* and *TLR4*, in a total volume of 25 *μl* (2 *μl* cDNA sample, 1 *μl* of each primer, 12.5 *μl* of SYBR Green PCR Master Mix and 8.5 *μl* of injected distilled water was used. GAPDH was used as an internal control. The quantitative real-time PCR conditions were as follows: 95*°C* for 15 *m*in (95*°C* for 15 *s*, 60*°C* for 30 *s*, 72*°C* for 30 *s*) for 40 cycles. Relative *TLR2* and *TLR4* expressions were normalized by GAPDH and fold changes were calculated using 2^−ΔΔCt^ method. The sequences of these primers are as follows:

TLR2, F: 5′-AAGAGGAAGCCCAAGAAAGC-3′
TLR2, R: 5′-CGATGGAATCGATGATGTTG-3′
TLR4, F: 5′-ACCTGGCTGGTTTACACGTC-3′
TLR4, R: 5′-CTGCCAGAGACATTGCAGAA-3′
GAPDH, F: 5′-ATGGACTGTGGTCATGAGCC-3′
GAPDH, R: 5′-ATTGTCAGCAATGCATCCTG-3′

### Lesion measurement

Lesion size was measured weekly in the rest of mice in each group. Measurement was conducted using caliper ruler for 7 weeks.

### Statistical analysis

Real time PCR was performed in triplicates. The results were calculated as mean±SD. To compare values, p-values of <0.05 were considered significant. All analysis was performed using Graph Pad Prism (Version 5) and online site (Pcr data analysis. sabiosciences. com). At first, Kolmogorov-Smirnov test was done and after normal distribution for our data, one way ANOVA was conducted.

## Results

**Figure 1.**
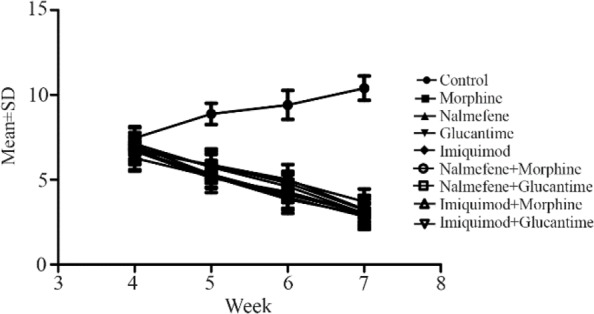
Mean and SD of expression of *TLR2* (A) and *TLR4* (B) as a relative fold change by real time PCR in lesions of infected mice compared to control by using ΔΔCt method normalized with GAPDH Control (Infected mice), M (1 *mg/kg* morphine), N (1 *mg/kg* nalmefene), G (20 *mg/kg* glucantime), I (Cream 5% imiquimod), M+N (1 *mg/kg* morphine+1 *mg/kg* nalmefene), N+G (1 *mg/kg* nalmefene+20 *mg/kg* glucantime), I+M (Cream 5% imiquimod+1 *mg/kg* morphine) ^*^(p<0.05), ^**^(p<0.01).

The results were normalized based on the internal control gene of GAPDH. They show that increase of expression of *TLR2* gene relative to the control group is apparent among all groups, and the difference was significant (p<0.05). The highest level expression of *TLR2* was seen in the groups treated by glucantime, imiquimod and imiquimod plus morphine, respectively. The expression of *TLR4* gene was reduced in groups under treatment with morphine and morphine plus nalmefene relative to the control group. The difference was meaningful in all groups, except in imiquimod plus glucantime group, despite expression increase. The highest expression was seen in the group treated by glucantime (p=0.00 11; mean=14.682) followed by the groups treated by imiquimod and imiquimod plus morphine, (p=0.0022, p=0.0073) respectively (Figure 1). The expression of *TLR2* and *TLR4* in morphine and glucantime groups in comparison with control group showed significant difference (p<0.01).

**Chart 1.**
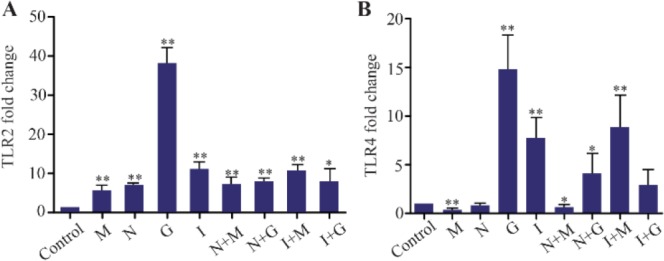
The mean±SD lesion in the test and control groups after treating mice with M (Morphine), N (Nalmefene), G (Glucantime), I (Imiquimod) during 7 weeks after challenge with promastigotes of *Leishmania major* in stationary phase.

The mean lesion size decreased in all treated groups and the differences with control group were significant (p<0.05). However, in this study, it was found that despite decreasing the size of lesion in all treatment groups, expression of *TLR4* in the morphine, nalmefene, morphine plus nalmefene treated groups compared to the control group was decreased. In all of the groups, the reduction in lesion size was observed in the control group. The mean lesion size in the control groups was 7.46 *mm* after challenge; at the end of week 7, the mice lesion size reached 10.4 *mm*. All treated groups showed a significant difference in comparison with the control group after 6 weeks (p<0.05, Table 1, Chart 1).

**Table 1.** The mean±SD lesion in the test and control groups after treating mice with M (Morphine), N (Nalmefene), G (Glucantime), I (Imiquimod) during 7 weeks after challenge with promastigotes of *Leishmania major* in stationary phase

**Group week**	**Control Mean±SD**	**M Mean±SD**	**N Mean±SD**	**G Mean±SD**	**I Mean±SD**	**N+M Mean±SD**	**N+G Mean±SD**	**I+ M Mean±SD**	**I+G Mean±SD**
**4**	7.46±0.61	6.92±0.68	6.72±0.76	6.68±1.08	7.12±1.01	6.74±0.84	7±0.78	6.76±0.68	6.28±0.77
**5**	8.88±0.63	5.78±0.71	5.88±0.68	5.11±.87	5.74±0.95	5.86±0.96	5.32±0.81	5.26±0.74	5.19±0.64
**6**	9.41±0.86	4.93±0.56	5.02±0.87	4.28±1.01	4.61±0.76	4.81±0.63	4.12±0.91	3.96±0.64	3.85±0.83
**7**	10.4±0.72	3.71±0.75	3.25±0.74	3.05±0.98	3.02±0.82	3.22±0.86	3.05±0.97	2.84±0.73	2.96±0.69

## Discussion

Macrophages are necessary for the Leishmaniasis survival, reproduction and differentiation. Immune systems are suppressed by drug addiction so the host will be sensitive to infectious disease 10. Concerning the TRLs signaling, immunity response with intermediate has important TRL4 in clinical consequences. Inhibition of *TRL2* expression in animal media showed that apoptosis decreases seriously in microglia cells produced by virus infection. Earlier studies have suggested that TRL2 deficiency in BALB/c mice reduces microglia function considerably, after long term morphine treatment 11. The findings revealed that opioids are targets for activation of microglia through TLR2. C57bl/6 mice lacking TLR2 are resistant to infection with *L. major* 12. In this study, the morphine treated group had the lowest level of *TRL2* expression, among the drug treated groups. Despite lesion size reduction in the glucantime treated group, the *TRL2* expression was the highest for an unknown reason. Concerning the TRL2, all groups treated by various drugs had increased expression, relative to the control group. Toluei *et al* also found similar results in human lesions 13. The highest level of expression belongs to the glucantime treated group (p<0.0001). Other groups had also meaningful difference with the control group (p< 0.001), except for the imiquimod plus glucantime treated group (p=0.0133). TLR4 has a controlling role in infection with *L. major* whose increase of expression reduces the lesion. The results show that *TLR2* increases expression in all groups compared with the control group and the difference between them is significant.

## Conclusion

Therefore, morphine may have a different function mechanism in treatment of the infection with the *L. major*. Morphine increases the expression of *TLR2* and decreases *TLR4* whereas imiquimod increases the expression of both *TLR2* and *TLR4*. The results of combination of morphine plus imiquimod were similar toimiquimod alone. At the end, it is suggested that the expression of other TLRs also be evaluated in the mice's lesion.
